# Editorial: Mechanisms, markers and therapeutics of synaptic pathology in Alzheimer's disease

**DOI:** 10.3389/fnins.2022.1022447

**Published:** 2022-10-06

**Authors:** Athanasios Metaxas, Bente Finsen

**Affiliations:** ^1^Department of Life Sciences, European University Cyprus, Nicosia, Cyprus; ^2^Department of Molecular Medicine, University of Southern Denmark, Odense, Denmark

**Keywords:** synapses, Alzheimer's disease models, ApoE, epigenetics, immunotherapy, lipid metabolism, microglia, astrocytes

Alzheimer's disease (AD) is a terminal neurocognitive disorder, and a leading cause of disability and dependency among elderly people globally. The disease has an insidious onset and is characterized by gradual impairment in one or more cognitive domains, most notably learning and memory, language, attention, and executive function. To date, synapse loss has been established as the strongest pathological correlate of the progressive cognitive decline of AD, indicating that deficiency in the structural and possibly functional integrity of the synapses is central to the disease process. In this Research Topic, we present recent findings regarding the drivers of synaptic loss in AD. We further consider potential neuroprotective therapeutic approaches, and discuss the hurdles associated with translating them from the laboratory to the clinic.

Amyloid-β and tau constitute the defining histopathological features of AD. Sanchez-Varo et al. show that synaptic pathology is observed by 4 months of age in the *APP*_751SL_/*PS*1_M146L_ model of amyloidosis, and is accompanied by deficits in spatial memory, as measured in the Morris water maze test. By performing transmission electron microscopy and a detailed analysis of the ultrastructure of hippocampal synapses in the periphery of senile plaques, the authors demonstrate that a spatial relationship between amyloid deposition, the plaque-associated oligomeric halo, and synaptic impairment is established early in the life of this AD mouse model. Importantly, it is shown that Aβ-induced, behaviorally-relevant synaptic damage is not only restricted to a reduction in synaptic density, but includes the depletion of synaptic vesicles in the periplaque presynaptic terminals, a finding with potential functional and therapeutic significance. Somogyi and Wolf employ a multi-compartmental computational modeling approach to assess the impact of tau pathology on the electrophysiological properties of neocortical layer III neurons in 9-month-old transgenic rTg4510 mice. Three-dimensionally reconstructed morphological and electrophysiological data were employed to inform the neuronal models. The authors predict that intraneuronal signal propagation slows down in tau mutant neurons, which may potentially lead to a general slow-down in signaling of the affected neural networks. However, neither the resistance and capacitance of the neuronal membrane, nor the integration of multiple incoming signals are altered by the mutant tau protein. These observations suggest that tau pathology may primarily be associated with failures/loss of synaptic connections at the network level, rather than intrinsic signaling alterations within individual neurons.

AD is increasingly being recognized as a heterogeneous disorder that involves several different etiological mechanisms, in addition to Aβ and tau. Konings et al. examine the effects of apolipoprotein E4 (ApoE4), a major genetic risk factor for sporadic AD, on synapses and neuronal activity, comparing them to the effects of the most common ApoE isoform, ApoE3. To determine the cell-source-specific effects of astrocytic and neuronal ApoE on neuronal excitability and binding to synaptic terminals, the authors employ primary mouse astrocytic and neuronal cultures and conditioned media from wild-type, *APOE*^ε3^ knock-in (KI), *APOE*^ε4^ KI and *APOE* knockout (KO) mice. It is demonstrated that ApoE targets the synapses when added to cultured neurons, differentially inducing changes in their activity, depending on whether it is derived from neurons or astrocytes. In addition to shedding light on the detrimental effects of ApoE4 on synaptic function, these data uncover a potentially therapeutic role for astrocytic ApoE3, which is shown to block the enhanced activity of *APP*_swe_/*PSEN*1_de9_ derived neurons. Chouhan et al. use an ME7 prion-induced model of chronic neurodegeneration to examine the effects of infection with live *Salmonella typhimurium* on a plethora of synaptic markers and markers of microglia-mediated neuroinflammation. The authors demonstrate that a single bacterial challenge in prion-infected mice aggravates neuroinflammatory responses, and reduces gene transcription in various synaptic plasticity pathways, particularly those involving glutamate receptors. The loss of glutamate receptor expression is accompanied by behavioral deficits, as evidenced in the burrowing assay. These data provide mechanistic insights as to how low-grade infection can exacerbate synaptic pathology, acting as a risk factor for the earlier onset and/or progression of neurodegenerative disease.

Highlighting new areas of potential therapeutic intervention, Paasila et al. provide a comprehensive overview of the state of research into the complex interactions between microglia, lipid metabolism and synaptic pathology in AD, while Chen et al. review the epigenetic mechanisms of synaptic regulation in AD, focusing on DNA methylation, histone modification, and RNA interference. In both reviews, the disparities that exist between the results from animal model vs. human *post-mortem* or clinical studies are dully noted. Such disparities are comprehensively analyzed in Nimmo et al., who present a systematic, head-to-head comparison of the results obtained in experimental vs. clinical studies of amyloid-β and alpha-synuclein immunotherapy. The authors demonstrate that while neuropathological findings translate quite effectively from animal models to humans, the cognitive and functional outcome measures do not, and explore reasons for this disconnection.

Notwithstanding that caution is warranted when interpreting the outcome of studies performed in animal models of AD, the analysis presented in this Research Topic clearly indicates that there are multiple molecular and cellular mechanisms operating to damage the structural and functional integrity of the synapses throughout the course of AD. Understanding synapse loss in the complex environment of the AD brain may provide novel clues and targets for the development of neuroprotective therapeutic interventions.

## Author contributions

AM and BF contributed equally to the conception and organization of this Research Topic. Both authors approved the Editorial text accompanying the Research Topic.

## Conflict of interest

The authors declare that the research was conducted in the absence of any commercial or financial relationships that could be construed as a potential conflict of interest.

## Publisher's note

All claims expressed in this article are solely those of the authors and do not necessarily represent those of their affiliated organizations, or those of the publisher, the editors and the reviewers. Any product that may be evaluated in this article, or claim that may be made by its manufacturer, is not guaranteed or endorsed by the publisher.

